# An American Hospital

**Published:** 1895-04-13

**Authors:** Walt Whitman


### AN AMERICAN HOSPITAL.

By Walt Whitman.

Our army foiled with loss severe, and the sullen remnant retreating
Till after midnight glimmer upon us the lights of a dim-lighted building,
We come to an open space in the woods, and halt by the dim-lighted building.
'Tis a large old church at the crossing roads, now an impromptu hospital.
Entering but for a minute I see a sight beyond all the pictures and poems ever made,
Shadows of deepest, deepest black, just lit by moving candles and lamps,
And by one great pitchy torch stationary with wild red flame and clouds of smoke;
By these, crowds, groups of forms vaguely I see on the floor, some in the pews laid down.
At my feet more distinctly a soldier, a mere lad, in danger of bleeding to death (he is shot in the abdomen).
I stanch the blood temporarily (the youngster's face is white as a lily).
Then, before I depart, I sweep my eyes o'er the scene, fain to absorb it all.
Faces, varieties, postures beyond description, most in obscurity, some of them dead;
Surgeons operating, attendants holding lights, the smell of ether, the odour of blood,
The crowd, oh, the crowd of the bloody forms, the yard outside also filled,
Some on the bare ground, some on planks or stretchers, some in the death spasm sweating,
An occasional scream or cry, the doctor's shouted orders or calls,
The glisten of the steel instruments catching the glint of the torches.
These I resume as a chant. I see again the forms, I smell the odour,
Then hear outside the orders given, "Fall in, my men, fall in!"
But first I bend to the dying lad, his eyes open, a half smile gives he me;
Then the eyes close, calmly close, and I speed forth to the darkness,
Resuming marching, ever in darkness marching, on in the ranks,
The unknown road still marching.

